# Action Potential Modulation in CA1 Pyramidal Neuron Axons Facilitates OLM Interneuron Activation in Recurrent Inhibitory Microcircuits of Rat Hippocampus

**DOI:** 10.1371/journal.pone.0113124

**Published:** 2014-11-19

**Authors:** Sooyun Kim

**Affiliations:** IST Austria (Institute of Science and Technology Austria), Klosterneuburg, Austria; UCL School of Pharmacy, United Kingdom

## Abstract

Oriens-lacunosum moleculare (O-LM) interneurons in the CA1 region of the hippocampus play a key role in feedback inhibition and in the control of network activity. However, how these cells are efficiently activated in the network remains unclear. To address this question, I performed recordings from CA1 pyramidal neuron axons, the presynaptic fibers that provide feedback innervation of these interneurons. Two forms of axonal action potential (AP) modulation were identified. First, repetitive stimulation resulted in activity-dependent AP broadening. Broadening showed fast onset, with marked changes in AP shape following a single AP. Second, tonic depolarization in CA1 pyramidal neuron somata induced AP broadening in the axon, and depolarization-induced broadening summated with activity-dependent broadening. Outside-out patch recordings from CA1 pyramidal neuron axons revealed a high density of α-dendrotoxin (α-DTX)-sensitive, inactivating K^+^ channels, suggesting that K^+^ channel inactivation mechanistically contributes to AP broadening. To examine the functional consequences of axonal AP modulation for synaptic transmission, I performed paired recordings between synaptically connected CA1 pyramidal neurons and O-LM interneurons. CA1 pyramidal neuron–O-LM interneuron excitatory postsynaptic currents (EPSCs) showed facilitation during both repetitive stimulation and tonic depolarization of the presynaptic neuron. Both effects were mimicked and occluded by α-DTX, suggesting that they were mediated by K^+^ channel inactivation. Therefore, axonal AP modulation can greatly facilitate the activation of O-LM interneurons. In conclusion, modulation of AP shape in CA1 pyramidal neuron axons substantially enhances the efficacy of principal neuron–interneuron synapses, promoting the activation of O-LM interneurons in recurrent inhibitory microcircuits.

## Introduction

GABAergic interneurons play a key role in the control of activity, plasticity, and rhythmic activity in neuronal networks. A hallmark of GABAergic cells is their extreme diversity [1], which may suggest specialization for specific tasks in the circuit. One example for such a specialization is the somatostatin-expressing O-LM interneuron in the hippocampal CA1 region. This interneuron type is thought to be selectively involved in recurrent inhibition [2], since it receives excitatory input exclusively from pyramidal neurons and provides inhibitory output largely to the distal dendrites of CA1 pyramidal cells [2]–[4]. Thus, these cells form a canonical recurrent inhibitory microcircuit. It has been recently reported that O-LM interneurons fire high-frequency trains of APs in awake, behaving animals [5]. However, the cellular and synaptic mechanisms underlying the efficient activation of these interneurons remain unexplored. Both Ca^2+^-dependent facilitation of transmitter release from presynaptic terminals [6], [7] and active conductances in postsynaptic dendrites [8] may contribute to efficient interneuron activation. However, whether these mechanisms are sufficient to trigger recurrent inhibition remains unclear.

An alternative or additional mechanism that may contribute to the activation of O-LM interneurons is modulation of axonal AP shape [9]–[15]. Two types of axonal AP modulation were previously reported. First, repetitive activity may induce broadening of axonal and presynaptic APs and subsequent enhancement of transmitter release [9]. Second, long depolarizations can propagate from cell bodies to presynaptic terminals, similarly leading to AP broadening and release enhancement (“static analog modulation”) [10], [11], [13], [14]. Additionally, depolarization propagated along the axon may directly affect transmitter release, for example by activation of presynaptic Ca^2+^ inflow [16]. However, it is not known whether activity-induced axonal AP broadening or static analog modulation is a general phenomenon that widely occurs in the brain, for example in the hippocampal CA1 region. Furthermore, the physiological significance of AP modulation remains unclear. Activity-dependent AP modulation in hippocampal mossy fiber axons requires a large number of APs to reach a significant extent [9]. Furthermore, static analog modulation requires that the distance between synaptically connected neurons is shorter than the length constant of the axon. This may be the case in cortical columns [10], but not necessarily in other circuits where long-range connections prevail.

To address the role of axonal AP modulation in inhibitory microcircuits of the hippocampus, synaptic transmission at the glutamatergic synapse between hippocampal CA1 pyramidal neurons and O-LM interneurons was examined. These interneurons, receiving exclusive input from CA1 pyramidal neurons, establish a canonical recurrent inhibitory microcircuit in the brain [2]–[4]. To address the possible role of the modulation, both dual axon–soma recordings and paired recordings between synaptically connected neurons were performed. Axonal AP modulation substantially increased transmitter release at principal neuron–interneuron synapses, promoting the activation of GABAergic interneurons in recurrent inhibitory microcircuits.

## Materials and Methods

### Ethics Statement

All experiments were carried out in accordance with national and institutional guidelines approved by the Austrian Bundesministerium für Wissenschaft and Forschung. This study was approved by IST Austria Tierschutzgremium. Rats were deeply anesthetized with isofluorane before surgery to minimize suffering.

### Slice preparation and maintenance

Hippocampal slices (thickness, 350–400 µm) were prepared from the brains of 17- to 22-day-old Wistar rats of either sex. The brain was rapidly isolated and sliced transversally in ice-cold sucrose-containing physiological saline using a vibratome (Leica VT1200). Slices were incubated in a maintenance chamber filled with sucrose-saline at ∼36°C for 30 min, and subsequently stored at room temperature. For experiments, slices were then placed into a recording chamber perfused with standard physiological saline. Recordings were performed at room temperature (∼22°C, range: 21–24°C).

### Electrophysiology

**Figure 1:**
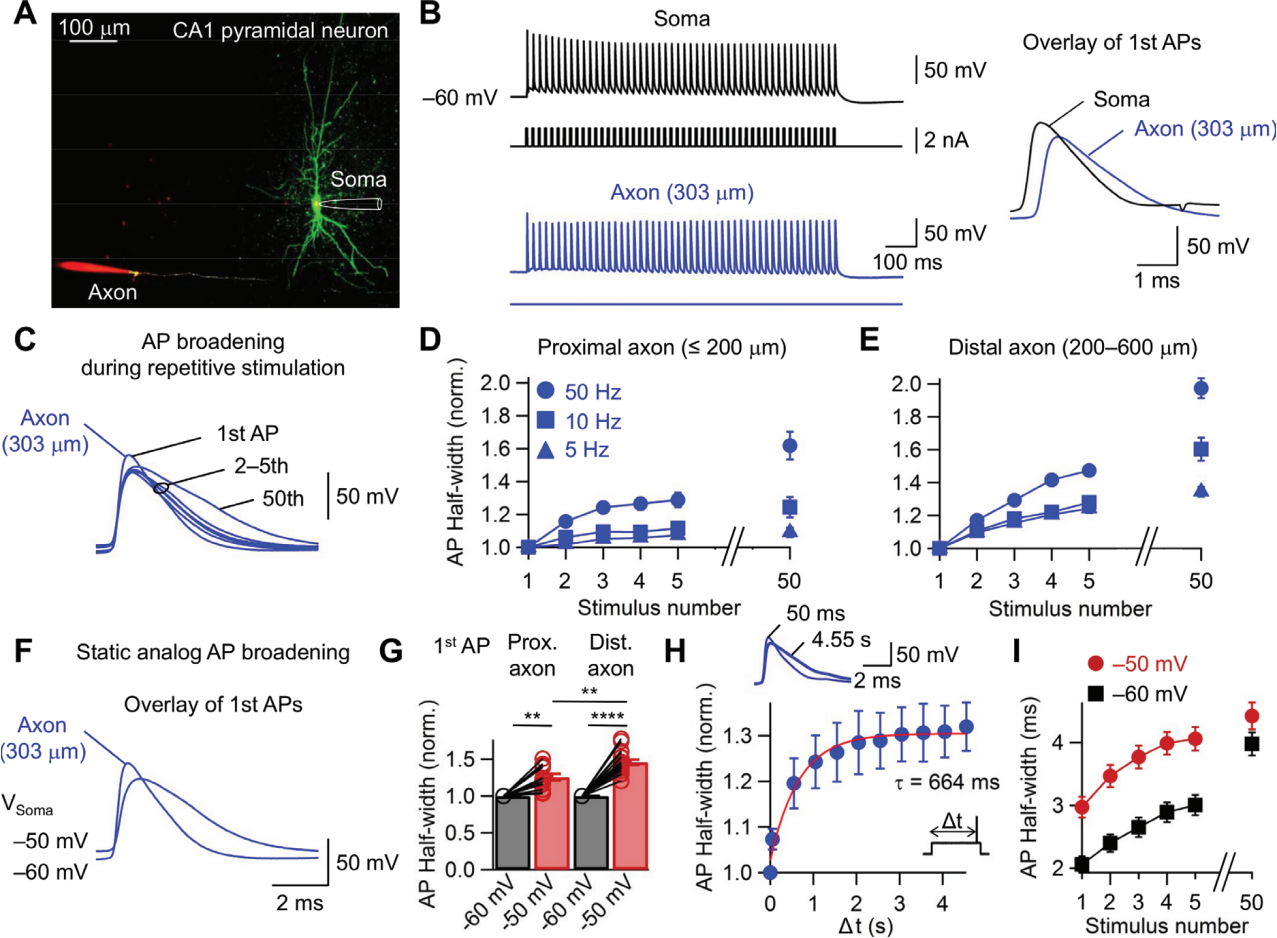
Activity-dependent broadening and static analog modulation of AP duration in axons of CA1 pyramidal neurons. (A) Axonal recording from a CA1 pyramidal neuron. Confocal stack maximum projection of a CA1 pyramidal neuron filled with 100 µM Alexa Fluor 488. The somatic recording pipette is illustrated schematically. Axonal recording pipettes contain 100 µM Alexa Fluor 647. (B) Left, APs evoked by a train of short pulses applied to the soma (5-ms duration, 1.5 nA amplitude, 50 Hz). Black traces indicate somatic voltage and corresponding current, blue traces represent axonal voltage and corresponding current. Right, traces show first action potential in the train on an expanded timescale. Axonal recording site is 303 µm from the soma. (C) Overlay of 1–5th, and 50th axonal AP in the train evoked by brief current pulses applied to the soma. The somatic membrane potential before and after train stimulation was –60 mV. Axonal recording site is 303 µm from the soma. (D, E) Plot of normalized AP half-duration against stimulus number for proximal (≤200 µm; D) and distal (200–600 µm; E) axonal recordings at 5 (triangles, n = 6, and 6, respectively), 10 (squares, n = 8, and 8, respectively), and 50 Hz (circles, n = 13, and 18, respectively) stimulus frequency. Note significant extent of single-trial broadening after the first AP. (F) Overlay of the first axonal AP evoked by a brief current pulse applied to the soma. The somatic membrane potential preceding the stimulation was –50, and –60, respectively. Axonal recording site is 303 µm from the soma. (G) Summary bar graph of axonal AP durations at half-maximal amplitude of two different groups (–60 mV, black, or –50, red) pooled for proximal (≤200 µm, n = 13) and distal (200–600 µm, n = 18) axonal recordings. Bars indicate mean ± SEM; circles denote individual experiments. Data from the same experiment were connected by lines. **P<0.01, ***P<0.001, ****P<0.0001. (H) Axonal APs evoked at the soma 50 ms (thin) and 4.55 s (thick) after the onset of somatic depolarization (–50 mV). Plot of normalized AP half-width versus latency (Δt). Data fitted with a single exponential function (red line). Note the increase in AP half-width for long delays. (Inset) Stimulation protocol. Axonal recording site is ∼120 µm from the soma. (I) Plot of half-width of distal axonal AP against stimulus number. Data points for the same experimental conditions are connected by lines. Black squares, –60 mV; red circles, –50 mV. Error bars, SEM.

All recordings were performed in the hippocampal CA1 subfield. Recordings from axons of CA1 pyramidal neurons were made as described previously [17], [18]. First, a somatic whole cell recording was made, using an internal solution containing Alexa Fluor 488 (100 µM, Invitrogen). Second, after a ∼20 min dye-loading period, fluorescently labeled axons were traced from the CA1 pyramidal neuron soma into stratum oriens-alveus using a Nipkow spinning disk confocal microscope (Ultraview live cell imager, Perkin Elmer, equipped with an Orca camera, Hamamatsu, and an argon/krypton laser or solid state laser at a wavelength of 488 nm). Finally, fluorescent and infrared differential interference contrast (IR-DIC) images were compared and CA1 pyramidal neuron axon blebs were patched under IR-DIC [10]. Exposure times were minimized to avoid phototoxicity. Both pipettes were held in the current-clamp configuration, and the series resistance was 7–82 MΩ. Pipette capacitance and series resistance compensation (bridge balance) were used throughout current-clamp recordings. Bridge balance was checked repeatedly and readjusted as required. For illustration purposes (Fig. 1A), one axonal recording was scanned with a Leica TCS SP5 II confocal microscope (Leica Microsystems).

Paired recordings between synaptically connected CA1 pyramidal neurons and O-LM interneurons were obtained as follows. First, a somatic whole-cell recording was obtained from an O-LM interneuron. Interneurons were identified by the location of the cell body near the stratum oriens/alveus border and their horizontally extending dendrites. Identification was corroborated by the prominent membrane voltage “sag” in response to long hyperpolarizing current pulses [8], [19], [20]. Finally, a second somatic whole-cell recording was obtained from a CA1 pyramidal neuron. K*^+^*-rich solution leaking from the pipette allowed us to identify CA1 pyramidal neurons likely to be synaptically connected. First, the vicinity of the synaptically connected CA1 pyramidal neuron was searched using the pipette with moderate pressure (40–50 mbar) by monitoring the response of postsynaptic O-LM interneuron under voltage-clamp configuration. Thereafter, the synaptically connected CA1 pyramidal neuron was patched. All O-LM interneurons included in this study were confirmed by *post hoc* morphological analysis by identifying their horizontally spreading dendrites and the long axonal projection to the stratum lacunosum-moleculare. The presynaptic CA1 pyramidal neuron was held in the current-clamp configuration; the postsynaptic O-LM interneuron was recorded in the voltage-clamp configuration at a membrane potential of –70 mV throughout. Series resistance in the postsynaptic recording (7–20 MΩ) was not compensated, but carefully monitored during the experiment using short 5 mV pulses following the evoked EPSC.

Outside-out patch recordings were obtained from CA1 pyramidal neuron somata or axons. Recordings were made from either conventional outside-out patch recordings or, in the case of axon recordings, isolated blebs [21]. In the latter case pipettes were retracted diagonally with moderate suction, using continuous visual control and decrease/increase of pressure as required (–30–100 mbar). Patches were held at –90 mV throughout. To measure voltage-gated K^+^ current, a pulse sequence consisting of a 100-ms prepulse to –120 mV and a 200-ms test pulse to 50 mV was generated. Voltage protocols were applied to outside-out patches once every ∼5 s.

Patch pipettes (open tip resistance 2–6 MΩ for somatic and 7–13 MΩ for axonal recording) were pulled from borosilicate glass tubing (outer diameter: 2 mm, inner diameter: 1 mm) with a horizontal pipette puller (P-97 or P-1000, Sutter Instruments). For outside-out patch recording, pipettes were coated with dental wax (Pluradent). Current- and voltage-clamp recordings were performed with a Multiclamp 700B amplifier (Molecular Devices).

Signals were low-pass filtered at 10 kHz in current-clamp recordings and 4 or 10 kHz in voltage-clamp recordings and digitized at a sampling rate of 20 or 100 kHz with a CED power 1401 mk II interface (Cambridge Electronic Design). Pulse protocols were generated using custom-made data acquisition software (FPulse 3.33; U. Fröbe, Freiburg) running under Igor Pro 6.2.2.2 (WaveMetrics). Leak and capacitive currents were subtracted online using a “P over –8” or “P over –4” correction procedure.

### Solutions and chemicals

The standard physiological saline solution contained 125 mM NaCl, 25 mM NaHCO_3_, 2.5 mM KCl, 1.25 mM NaH_2_PO_4_, 2 mM CaCl_2_, 1 mM MgCl_2_, and 25 mM D-glucose. The sucrose-containing physiological saline solution contained 87 mM NaCl and 75 mM sucrose, with 10 mM D-glucose. Both solutions were equilibrated with 95% O_2_ and 5% CO_2_ gas mixture. The K^+^-rich internal solution for whole-cell recording contained 120 mM K-gluconate and 10 mM EGTA, 20 mM KCl, 2 mM MgCl_2_, 0.2% biocytin, 10 mM HEPES, and 2 mM Na_2_ATP or 4 mM Na_2_ATP and 0.3 mM Na_2_GTP, pH adjusted to 7.3 with KOH. 100 µM Alexa Fluor 488 was added to the solution used for somatic recording. For paired recordings, K-gluconate and EGTA concentrations were modified to 135 mM and 0.1 mM, respectively. α-Dendrotoxin (α-DTX) was purchased from Alomone labs. For experiments with α-DTX, 0.1% bovine serum albumin (BSA) was added to the extracellular solutions to prevent peptide adsorption.

### Post-hoc morphological analysis

For *post hoc* morphological analysis, slices were fixed overnight in 2.5% paraformaldehyde, 1.25% glutaraldehyde, and 15% picric acid in 100 mM phosphate buffer (PB), pH 7.3. After fixation, slices were incubated in 1% hydrogen peroxide and shock-frozen in liquid nitrogen. Subsequently, the tissue was treated with PB containing 1% avidin-biotinylated horseradish peroxidase complex (ABC; Vector Laboratories) overnight at 4°C. Excess ABC was removed by several rinses with PB, before development with 0.05% 3,3′-diaminobenzidine tetrahydrochloride and 0.01% hydrogen peroxide. Finally, slices were rinsed in PB several times and embedded in Mowiol (Höchst). Distances were measured from the point of origin of the axon to the axonal recording site along the axonal trajectory (BX61 microscope, Olympus).

### Data analysis

Data analysis was performed using Igor Pro (Wavemetrics) or Stimfit [22]. AP duration was measured at half-maximal amplitude, using threshold and peak as reference points. AP threshold was defined as the time point at which dV/dt≥20–50 V s^–1^. To quantify short-term dynamics of synaptic transmission, traces were averaged and the peak amplitude of each EPSC in a train was measured from the baseline directly preceding the rising phase. For calculation of conductance, reversal potential for K^+^ currents was assumed –95 mV [23]. Membrane potentials are given without correction for liquid junction potentials.

Values indicate mean ± SEM values. Error bars in the figures also represent the SEM. Significance of differences of mean values was assessed by a two-sided nonparametric Wilcoxon signed rank test, or Wilcoxon rank sum test.

## Results

### Modulation of AP shape in CA1 pyramidal neuron axons

Modulation of axonal AP shape in CNS neurons has been described in hippocampal mossy fiber axons, hippocampal CA3 pyramidal neuron axons and neocortical layer 5 pyramidal neuron axons [9]–[11], [14]. Whether AP modulation also occurs in other types of axons has remained unclear. To address this question, simultaneous axon–soma recordings from CA1 pyramidal neurons in hippocampal slices were made (Fig. 1). Axonal recordings were established in three steps. First, a somatic recording was obtained with a patch pipette containing Alexa Fluor 488. Second, the axon was visualized using fast confocal imaging after sufficient filling time. Finally, recordings were made from axon blebs [10], [17], [18].

First, I tested for activity-dependent modulation of AP shape [9]. Trains of high-frequency stimuli (5, 10, and 50 Hz) were applied at the soma, and AP half-duration in the axon was plotted against stimulus number (Fig. 1B–E). Intriguingly, the half-duration of the AP in CA1 pyramidal neuron axons broadened substantially during repetitive stimulation. AP broadening was frequency dependent, with an extent that was minimal at 5 Hz and maximal at 50 Hz (Fig. 1D, E). Furthermore, AP broadening was more pronounced in distal (200–600 µm) than in proximal axonal sites (≤200 µm; P<0.05). Thus, these results suggest that CA1 pyramidal neuron axons, like mossy fiber axons [9], show activity-dependent broadening of AP shape. However, the onset of broadening in CA1 pyramidal neurons was surprisingly rapid. Significant AP broadening was already observed following a single AP, and slow increase in AP half-width was shown after five APs. For a 50 Hz train, the initial rate of broadening onset at distal axonal sites was τ = 11% AP^–1^. Thus, the onset of AP broadening in CA1 pyramidal neuron axons was surprisingly fast, substantially more rapid than in hippocampal mossy fiber axons [9].

To examine whether CA1 pyramidal neuron axons show static analog modulation of AP shape by somatic depolarization [10], the presynaptic holding potential was set to –60 mV, or –50 mV, and short suprathreshold current pulses were applied at the soma to initiate APs (Fig. 1F–I). Depolarization at the soma led to substantial AP broadening in the axon in comparison to the control value at –60 mV (Fig. 1G; P<0.0001, n = 31). Thus, CA1 pyramidal neuron axons, similar to layer 5 neocortical pyramidal neuron axons [10], showed substantial static analog modulation of AP shape. Static AP broadening was more prominent in distal than in proximal axons (P<0.01). To examine the time course of static analog modulation of AP shape, AP was elicited at different time points after the onset of somatic depolarization (Fig. 1H; –50 mV). The time course of depolarization-induced axonal AP broadening was fitted with a single exponential function with a time constant of 664 ms (Fig. 1H; 74–597 µm, n = 8). Finally, I examined the interaction of high-frequency activity and static AP broadening (Fig. 1I). Whereas five APs evoked at a frequency of 50 Hz increased the duration of the AP by 147%, and depolarization to –50 mV broadened the axonal AP by 146%, the combination of both stimuli resulted in a broadening by 201% in comparison to control values (Fig. 1I). In conclusion, our results demonstrate that AP modulation in CA1 pyramidal neuron axons is very prominent. In combination, high-frequency stimulation and static analog modulation can broaden the axonal AP by up to two-fold.

### Mechanisms of axonal AP modulation

**Figure 2:**
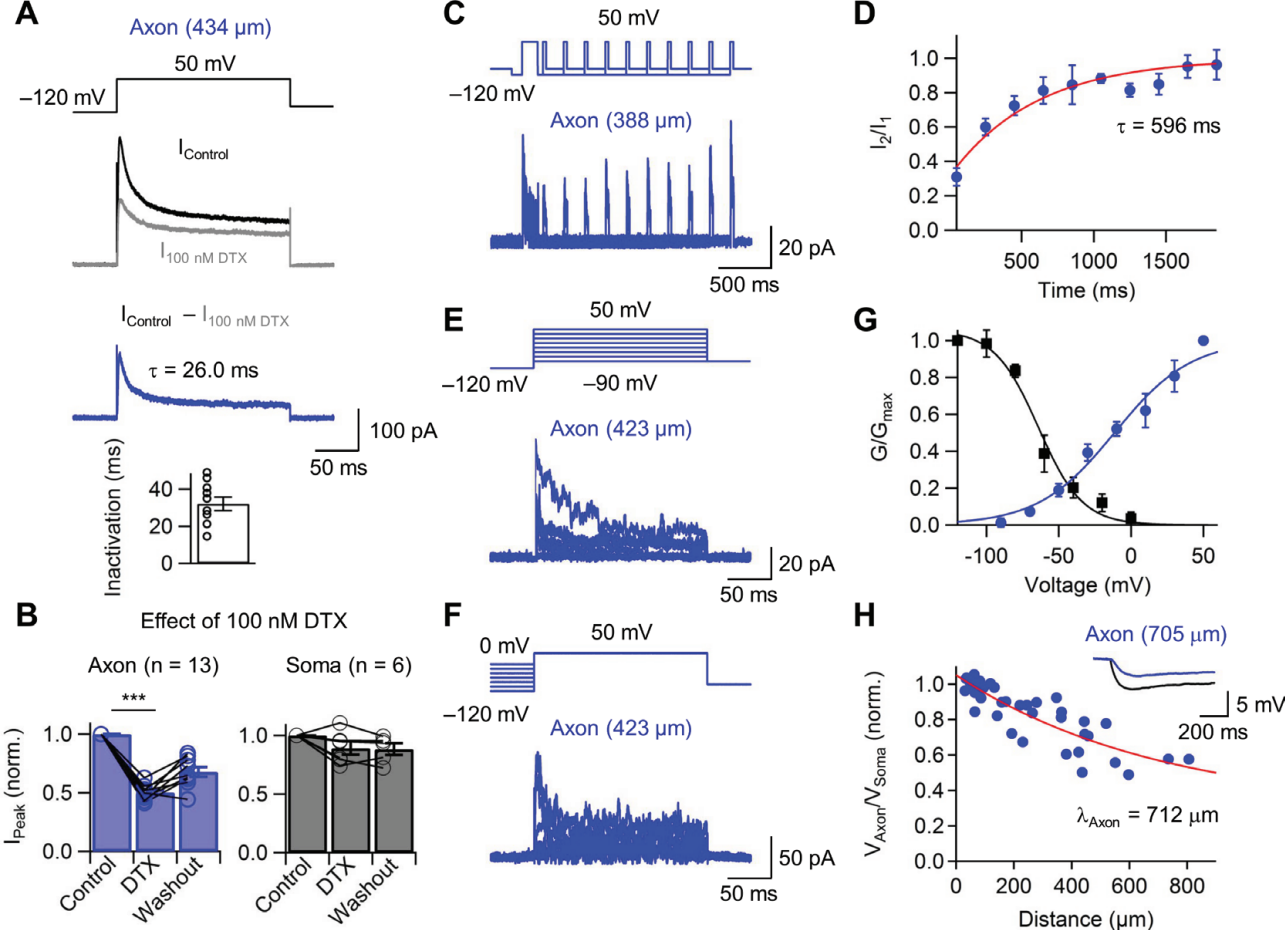
Inactivating axonal Kv1 channels and long length constant of the axonal cable are the key mechanisms underlying axonal AP modulation. (A) Top, K^+^ current evoked in outside-out patches from the axon (434 µm) of CA1 pyramidal neuron by 200-ms pulses to 50 mV in the absence (Black trace, I_Control_) and presence (Gray trace, I_100 nM α-DTX_) of 100 nM α-DTX. Middle, the blue trace represents the α-DTX-sensitive current component obtained by digital subtraction. Bottom, bar plot of the average time constant of inactivation (n = 10). (B) Summary bar graph showing the effect of 100 nM α-DTX in axonal patches (left, P<0.001, n = 13) and somatic patches (right, P>0.1, n = 6). Bars indicate mean ± SEM; circles denote individual experiments. Data points for the same experimental conditions are connected by lines. (C) Recovery of α-DTX-sensitive K^+^ channel from inactivation. Stimulation protocol and example traces of recovery from inactivation, as probed with a double-pulse protocol (100-ms prepulse to –120 mV, 150-ms conditioning pulses and 30-ms test pulses to +50 mV, separated by a recovery interval of variable duration at –120 mV). The holding potential before and after the pulse protocol was –90 mV. Axonal patch is 388 µm from the soma. (D) Summary plot of the amplitude of the peak current evoked by the test pulse, divided by that evoked by the conditioning pulse, plotted against interpulse interval. Data points represent means from 5 patches. Red curves represent double-exponential fit to the data points. (E, F) Gating properties of α-DTX-sensitive axonal K^+^ channels. Stimulation protocol and traces of activation and steady-state inactivation of α-DTX-sensitive K^+^ channels in CA1 pyramidal neuron axons. (E) To probe steady-state activation, a 100-ms prepulse to –120 mV was followed by a 200-ms test pulse to various potentials (–120 to 50 mV). (F) To test steady-state inactivation, a 5-s prepulse to various potentials (–120 to 0 mV) was followed by a 200-ms test pulse to 50 mV. Axonal patch is 423 µm from the soma. (G) Activation (blue circles) and steady-state inactivation (black squares) curves. Conductance values were normalized to the maximal value. Data points represent means from 8 patches for activation curve and 6 patches for steady-state inactivation. Blue curves represent Boltzmann functions fit to the data points. For the activation curve (blue line), the midpoint potential was –12 mV and the sloe factor 26.4 mV. For the inactivation curve (black line), the midpoint potential was –64 mV and the slope factor 15.2 mV. (H) Length constant of the CA1 pyramidal neuron axons. Plot of axonal to somatic voltage deflection during hyperpolarizing current pulses (1 s, –30 pA) applied at the soma, plotted against distance. Each data point represents a simultaneous axon–soma recording. Red line represents a fit of data points with an exponential function, resulting in a mean length constant of 712 µm. Note that the long length constant results in particularly efficient propagation of subthreshold membrane potential changes from the soma to the axon. (Inset) voltage changes recorded in the soma (black) and the axon (blue) during hyperpolarizing current pulses applied at the soma. Axonal recording site is 705 µm from the soma. Traces shown represent average of 20 (A, black trace), 22 (A, gray trace), 2 (C), 3 (E), and 2 (F) single sweeps. Transient inward Na^+^ currents at the beginning of the pulse are truncated. Error bars, SEM.

What are the mechanisms underlying activity-dependent AP broadening and static AP broadening in the axon (Fig. 2)? Previous studies indicated that inactivation of axonal and presynaptic Kv1 channels plays a key role in broadening [9], [24]. To examine this, I directly probed the presence of Kv1 channels in CA1 pyramidal neuron axons by the specific blocker α-DTX, which blocks Kv channels assembled from Kv1.1, Kv1.2, and Kv1.6 subunits. Whereas α-DTX-sensitive channels were nearly absent in the somatic membrane, 100 nM α-DTX blocked a substantial portion of the peak K^+^ current in outside-out patches or blebs isolated from CA1 pyramidal neuron axons (Fig. 2A, B; axon (111–618 µm), 51±1.9%, P<0.001, n = 13; soma, 89±5.5%, P>0.1, n = 6). Digital subtraction of K^+^ current traces in the presence of 100 nM α-DTX from those under control conditions revealed that the α-DTX-sensitive current component was rapidly inactivating (Fig. 2A). On average, the inactivation time constant was 32.1±3.6 ms (Fig. 2A; 10 outside-out patches and isolated blebs). Thus, whereas Kv1 channels are absent from cell bodies (see [23]), they are expressed in high density in the axons of CA1 pyramidal neurons.

To examine whether the gating properties of Kv1 channels in CA1 pyramidal neuron axons are consistent with a possible involvement of these channels in activity-dependent AP broadening, the time course of recovery of K^+^ channels from inactivation was measured using a double-pulse protocol (Fig. 2C). Recovery of K^+^ channels from inactivation showed a prominent slow component, with time constant values of 596±83 ms (Fig. 2D; 111–434 µm; 5 isolated blebs). Taken together, the fast onset of inactivation and the slow recovery suggest that axonal Kv1 channels have gating properties that are highly suitable for activity-dependent AP broadening.

To test whether the gating properties of Kv1 channels in CA1 pyramidal neuron axons will also support static AP broadening, steady-state activation and inactivation curves were measured using pulse protocols with varying prepulse and test pulse amplitudes (Fig. 2E, F; 8 and 5 isolated blebs for activation and inactivation curve, respectively). Interestingly, steady-state inactivation of axonal K^+^ channels occurred in a membrane potential region close to the resting potential of CA1 pyramidal neurons (Fig. 2G). The steep region of the inactivation curves was highly overlapping with the resting potential values of CA1 pyramidal neurons under in vitro and in vivo conditions [25], [26].

The second requirement for static analog modulation is a long length constant of the axonal cable [10], [11], [14]. To measure the length constant of the axonal cable in CA1 pyramidal neurons, long hyperpolarizing current pulses were applied at the soma, and the degree of attenuation as the ratio of axonal over somatic voltage changes was quantified (Fig. 2H). Fitting the data with an exponential function revealed a length constant λ of 712 µm (40 simultaneous axon–soma recordings). This long length constant of the axonal cable is consistent with the idea of analog modulation of axonal APs by somatic depolarization. To corroborate the effect of a long length constant of the axonal cable on AP modulation, the spatial property of depolarization-induced analog modulation of axonal APs was tested (Fig. S1). The extent of AP broadening gradually decreases as a function of distance from the soma with a distance constant of 883 µm (Fig. S1; 39 simultaneous axon–soma recordings). Taken together, the marked extent of steady state inactivation and the long length constant of the CA1 pyramidal neuron axon make downstream synapses particularly sensitive to static AP broadening.

### Axonal AP modulation promotes transmitter release at principal neuron–interneuron synapses

**Figure 3:**
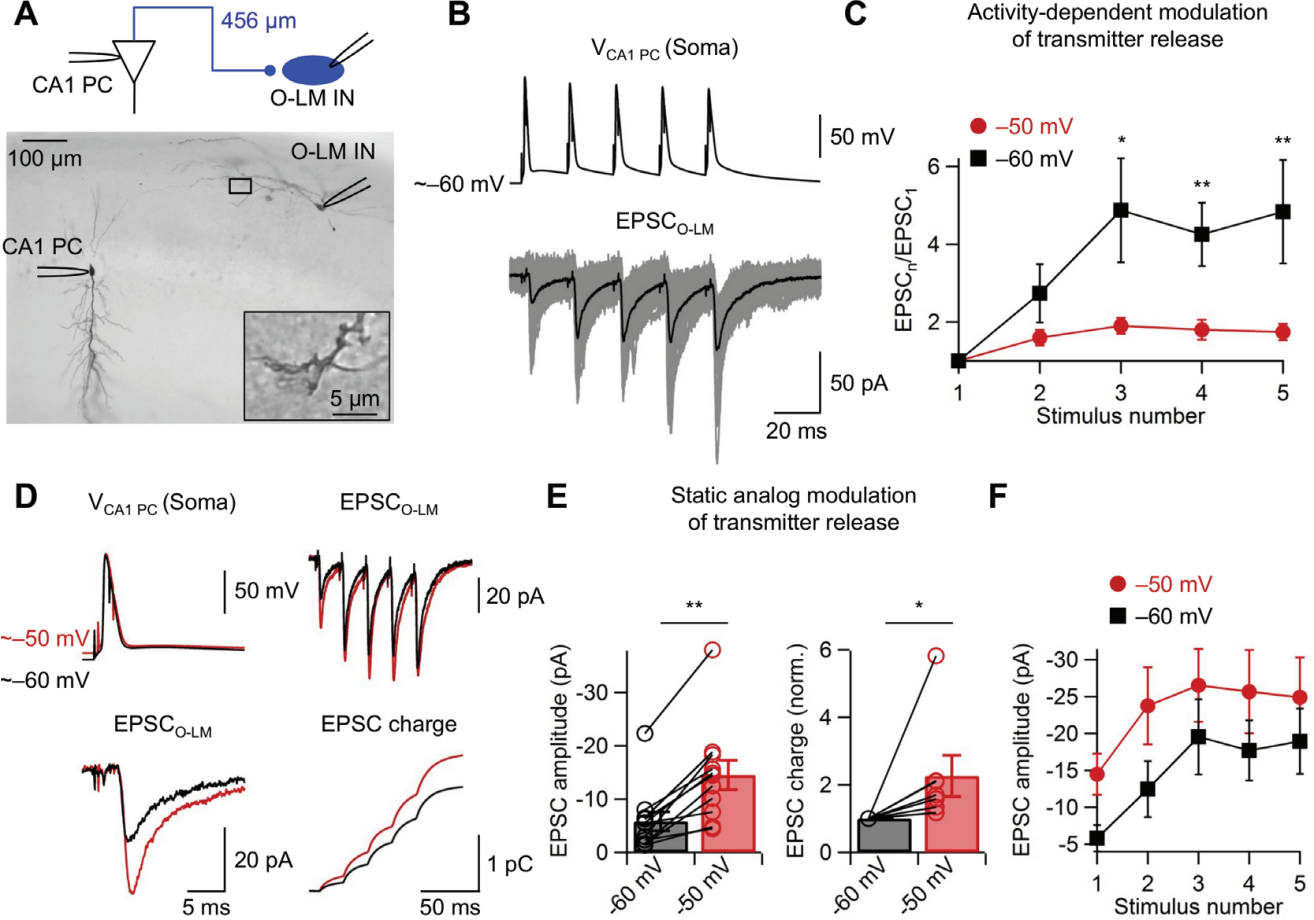
Modulation of synaptic transmission at CA1 pyramidal neuron (CA1 PN)–O-LM interneuron (O-LM IN) synapses. (A) Photomicrograph of a pair of a presynaptic CA1 pyramidal neuron and a postsynaptic O-LM interneuron filled with biocytin during recording. Inset shows putative synaptic contact at higher magnification. Inset, an expanded view corresponding to the dashed box indicates a putative synaptic contact which is 456 µm from CA1 pyramidal neuron soma. (B) Activity-dependent modulation of synaptic transmission. Paired recordings of presynaptic membrane potential (upper traces) and postsynaptic current (lower traces; 10 superimposed consecutive sweeps (gray), overlaid with the average (black)) for two different presynaptic holding potentials (red, –50 mV; black, –60 mV). Note the marked facilitation of EPSC peak amplitude. (C) Facilitation ratio (EPSC_n_/EPSC_1_; see Materials and Methods), plotted against stimulus number, for two different presynaptic holding potentials (red, –50 mV; black, –60 mV). Data from 11 CA1 PN–O-LM IN pairs. Error bars, SEM. Data points for the same experimental conditions are connected by lines. Asterisks indicate a significant difference. (D) Static analog modulation of synaptic transmission. Left, paired recordings of presynaptic membrane potential (upper traces) and postsynaptic current (lower traces) for single AP for two different presynaptic holding potentials (red, –50 mV; black, –60 mV). Right, postsynaptic current for a train of 5 APs (upper traces) for two different presynaptic holding potentials (red, –50 mV; black, –60 mV) and corresponding EPSC integral (lower traces), presumably proportional to the total amount of transmitter release. (E) Summary bar graph of the effects of presynaptic holding potential on peak amplitude of first EPSCs (left; n = 11) and normalized EPSC integral for a train of presynaptic stimuli (5 APs at 50 Hz; right; n = 9). Bars indicate mean ± SEM; circles denote individual experiments. Data from the same experiment were connected by lines. Note that changes in presynaptic membrane potential alter both the amplitude of first EPSCs (left) and the charge of the EPSC train (right). (F) Plot of EPSC peak amplitudes against stimulus number. Data points for the same experimental conditions are connected by lines. Black squares, –60 mV; red circles, –50 mV. Error bars, SEM. *0.01≤P<0.05, **P<0.01.

To examine the functional consequences of modulation of axonal APs, the properties of synaptic transmission at synapses between CA1 pyramidal neuron axons and O-LM interneurons in the hippocampal CA1 region were tested (Fig. 3). To obtain information about the length of the axonal trajectory, paired recordings with pre- and postsynaptic biocytin labeling were combined (Fig. 3A; average axonal length from the point of origin of the axon to putative synaptic contact: 296±26 µm, n = 10). EPSCs evoked by trains of five presynaptic stimuli at a frequency of 50 Hz showed marked facilitation (Fig. 3B, C). Likewise, somatic depolarization in the presynaptic CA1 pyramidal neuron induced substantial enhancement of first EPSC amplitude (Fig. 3D; P<0.001, n = 11). In combination, activity-dependent facilitation and facilitation evoked by somatic depolarization increased the integral of 5 EPSCs by 2.3±0.6 fold (Fig. 3E; 7 CA1 pyramidal neuron–O-LM interneuron pairs, P<0.05, n = 7). I tested the interaction of activity-dependent modulation and static modulation of synaptic transmission (Fig. 3F). Five presynaptic stimuli at a frequency of 50 Hz and somatic depolarization increased EPSC amplitude by 5-fold (Fig. 3F; EPSC_1_ at –60 mV: –5.81±1.80 mV; EPSC_5_ at –50 mV: –24.92±5.40 pA, n = 11). Thus, both factors substantially increased the efficacy of principal neuron–interneuron synapses.

**Figure 4:**
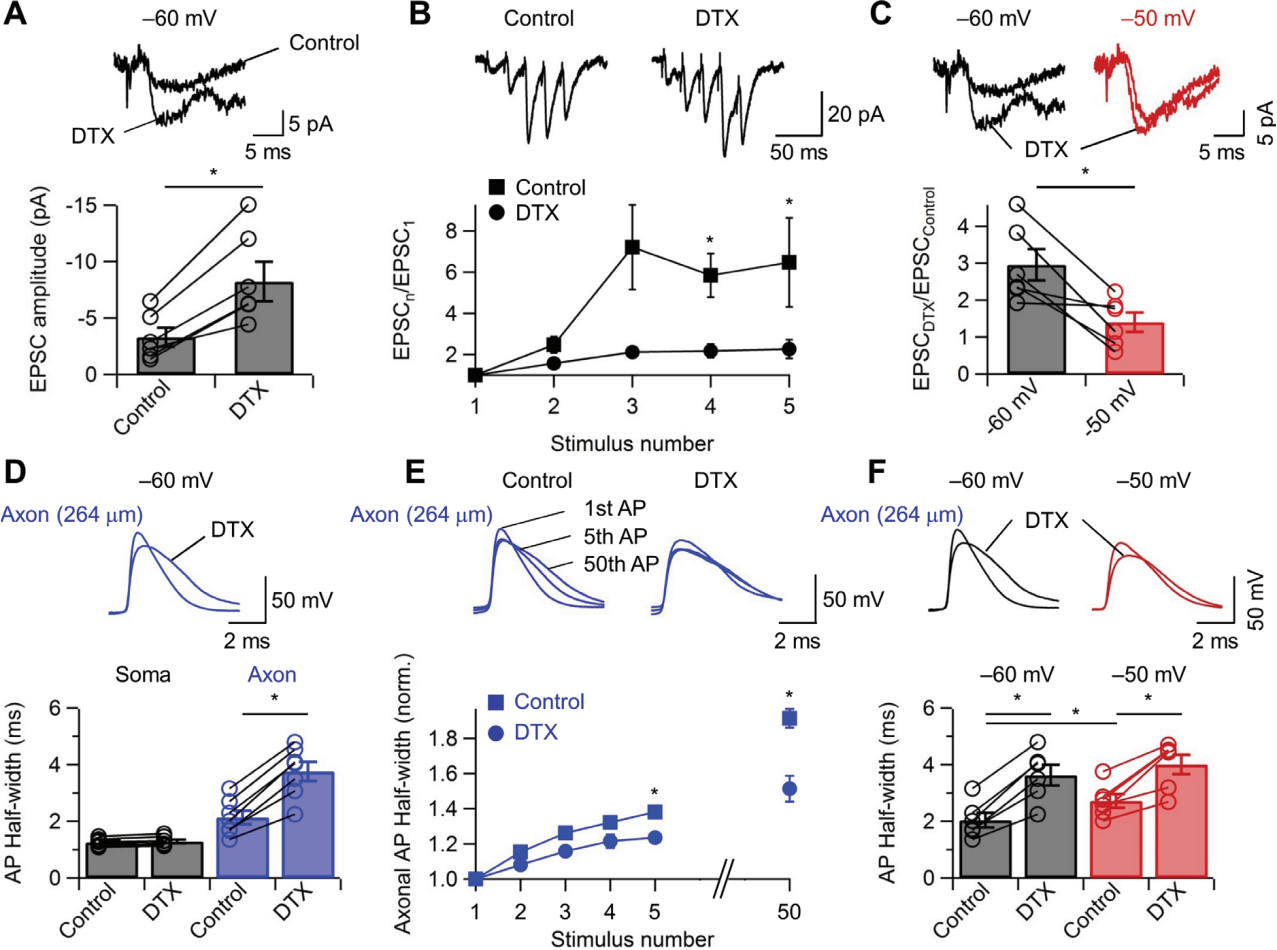
Modulation of synaptic transmission at CA1 pyramidal neuron–O-LM interneuron synapses is primarily controlled by axonal Kv1 channels. (A) Block of Kv1 channels enhances transmission. Top, unitary EPSCs at CA1 PN–O-LM IN synapses evoked by single presynaptic APs in control conditions and in the presence of 100 nM α-DTX. Traces represent averages from 10 single sweeps. Bottom, summary bar graph of the effects of α-DTX on EPSC peak amplitude. Note that α-DTX markedly increased synaptic efficacy. (B) Block of Kv1 channels reduces facilitation of transmission. Top, EPSCs at CA1 PN–O-LM IN synapses evoked by trains of five presynaptic APs in control conditions (left) and in the presence of 100 nM α-DTX (right). Bottom, facilitation ratio (EPSC_n_/EPSC_1_), plotted against stimulus number, in control conditions (squares) and in the presence of α-DTX (circles). Somatic holding potential –60 mV. (C) Block of Kv1 channels abolishes static analog modulation of transmission. Top, unitary EPSCs at CA1 PN–O-LM IN synapses evoked by single presynaptic APs in control conditions and in the presence of 100 nM α-DTX. Presynaptic membrane potential were held at –60 mV (left; same recording as in (A)), and –50 mV (right). Bottom, summary bar graph of the effects of α-DTX on EPSC peak amplitude (EPSC_DTX_/EPSC_Control_) for two different presynaptic holding potential (black, –60 mV; red, –50 mV). Note that α-DTX occluded the effects of changing membrane potential in the presynaptic neuron. (D) Block of Kv1 channels broadens the axonal AP. Top, axonal AP traces in control conditions and in the presence of 100 nM α-DTX. Bottom, summary bar graph showing the effects of α-DTX on half-duration of somatic and axonal AP. Axonal recording site is 264 µm from the soma. Note that α-DTX selectively increased axonal AP duration. (E) Block of Kv1 channels reduces activity-dependent AP broadening. Top, superposition of 1^st^, 5th, and 50^th^ axonal AP in control conditions (left) and in the presence of 100 nM α-DTX (right). Bottom, plot of axonal AP half-width against stimulus number in control conditions (squares) and in the presence of 100 nM α-DTX (circles). Somatic holding potential –60 mV. Data from 7 recordings at distances of 200 to 500 µm. Axonal recording site is 264 µm from the soma. (F) Block of Kv1 channels reduces static AP broadening. Top, superposition of axonal APs in control conditions and in the presence of 100 nM α-DTX at –60 mV (left; same recording as in (D)), and –50 mV (right). Bottom, summary bar graph of the effects of α-DTX on AP broadening at –60 mV (black) and –50 mV (red). Note that the depolarization-induced AP broadening reduced the effect of α-DTX. Axonal recording site is 264 µm from the soma. Bars indicate mean ± SEM. Open circles represent data from individual experiments. Data from the same experiment or for the same experimental conditions were connected by lines. *0.01≤P<0.05.

To further assess the contribution of Kv1 channels to the facilitation of transmitter release, the functional consequences of Kv1 channel block were investigated (Fig. 4A–C). First, I tested whether α-DTX would be able to mimic facilitation induced by either repetitive stimulation or somatic depolarization. On average, bath application of 100 nM α-DTX increased the amplitude of unitary EPSCs at CA1 pyramidal neuron–O-LM interneuron synapses by 224% (6 pairs, P<0.05). Second, I probed whether α-DTX occluded the effects of facilitation (Fig. 4B). Whereas facilitation was prominent under control conditions, it was markedly reduced in the presence of 100 nM α-DTX (Fig. 4B). This may suggest that α-DTX occluded facilitation generated by axonal and presynaptic AP broadening. Finally, whether α-DTX eliminated facilitation induced by somatic depolarization was examined (Fig. 4C). Conditioning depolarization of the presynaptic neuron significantly reduced the effect of α-DTX on the amplitude of unitary EPSCs at CA1 pyramidal neuron–O-LM interneuron synapses (Fig. 4C; P<0.05, n = 6).

To confirm that the observed effects on the amplitude of EPSCs were generated by AP broadening mediated by Kv1 channels, I further examined whether α-DTX mimicked and occluded AP broadening in axons of CA1 pyramidal neurons (Fig. 4D–F). 100 nM α-DTX led to significant increase of half duration of the axonal AP (Fig. 4D; P<0.05, n = 6), and further reduced the extent of activity-dependent AP broadening (Fig. 4E). Likewise, somatic depolarization significantly reduced the effect of α-DTX on axonal AP broadening (Fig. 4F; P<0.05, n = 6). Taken together, these results suggest that a substantial fraction of both mechanisms of facilitation are generated by axonal and presynaptic AP broadening.

## Discussion

The present paper reports the following major findings. First, our results identify two forms of AP modulation in CA1 pyramidal neuron axons. Substantial AP broadening in the axon can be induced by small number of APs and moderate depolarizations. Second, axonal AP modulation markedly enhances the efficacy of synaptic transmission at principal neuron–interneuron synapses in the hippocampal CA1 region. As O-LM interneurons are selectively involved in feedback inhibition [2], this suggests that axonal AP modulation promotes the recruitment of O-LM interneurons in recurrent inhibitory microcircuits.

### Two forms of AP modulation in CA1 pyramidal neuron axons

It is generally assumed that the AP is an all-or-none event with constant amplitude and time course, implying that the basic coding mechanism in the brain is digital [27]. However, recent evidence suggests that the axonal APs are subject to analog modulation in hippocampal mossy fiber axons [11] and axons of hippocampal CA3 pyramidal neurons [14] and layer 5 neocortical pyramidal neurons [10], [13], as originally found in invertebrates [28], [29]. Our results reveal two forms of AP modulation in CA1 pyramidal neuron axons: Activity-dependent AP broadening and static AP broadening. Whereas both phenomena have been reported previously in other axons, the extent of modulation in CA1 pyramidal neuron axons appears to be distinct from other cell types. In combination, both forms of modulation increase AP duration by a factor of 2 with physiological stimulation paradigms.

Activity-dependent AP broadening was first reported in hippocampal mossy fiber axons. In this axon type, broadening requires several hundred APs to reach a steady-state value [9]. As granule cells under in vivo conditions fire APs only sparsely [30], the physiological significance of AP broadening in this type of modulation is not entirely clear. In contrast, our results show that activity-dependent broadening in CA1 pyramidal neuron axons occurs after a single AP (single-trial broadening), followed by slow increase in broadening after ∼5 APs. In CA1 pyramidal neurons in vivo, bursts of action potentials are detected prominently during various behaviors [31]. Therefore, activity-dependent AP broadening is likely to occur under physiological conditions.

Static AP broadening was found in axons of layer 5 neocortical pyramidal neurons [10] and hippocampal CA3 pyramidal neurons [14]. A key requirement for this form of analog modulation is that the intercellular distance is smaller than the length constant of the axonal cable. In axons of layer 5 neocortical pyramidal neurons the length constant is ∼422–550 µm [10]. In comparison, the width of a cortical column is <500 µm. Thus, intra-columnar synaptic connections are likely to be affected by analog modulation. In contrast, in hippocampal mossy fiber axons, the distance between granule cell bodies and CA3 pyramidal neurons is often substantially larger than the length constant of the axon cable [11]. Therefore, tonic somatic depolarizations have small effects on the efficacy of synaptic transmission [11]. Our results show that AP duration is markedly affected by somatic depolarization. The length constant of the CA1 axon is 712 µm, suggesting that synaptic transmission will be affected on target cells in a large subregion of the hippocampal CA1 field.

### Mechanisms underlying axonal AP modulation

Our results suggest that AP broadening is mediated by inactivation of Kv1 channels. Several lines of evidence are consistent with this idea. First, CA1 pyramidal neuron axons express a high density of inactivating K^+^ channels [32]. These channels are blocked by α-DTX, suggesting that they are assembled from Kv1.1, 1.2, or 1.6 alpha subunits. Second, these channels inactivate rapidly, as required for activity-dependent AP broadening to occur. Third, these channels show marked steady-state inactivation, as required for static broadening. Finally, these channels appear to be present in the entire axon, but not in the soma [23]. Although the involvement of inactivating Kv1 channels in AP broadening is well established [24], the inactivation time constant seems particularly fast in CA1 pyramidal neuron axons. This could be due to higher expression levels of expression of Kv1.4 subunits, or beta subunits, or both [33], [34].

Our results also show that AP broadening makes a contribution to the facilitation of EPSCs during both repetitive stimulation and tonic somatic depolarization. Broadening of the presynaptic AP will have two effects. First, AP broadening may increase the open probability of presynaptic Ca^2+^ channels. Second, AP broadening will increase the duration of the Ca^2+^ current. Thus, although the driving force for Ca^2+^ ion flow may be slightly reduced, the total Ca^2+^ inflow will be increased [35]. Together with the biochemical cooperativity of the Ca^2+^ sensor [36], a small increase in Ca^2+^ inflow will generate substantial facilitation of transmitter release.

### AP modulation promotes activation of O-LM interneurons in recurrent inhibitory microcircuits

Surprisingly, it has been suggested that dendritic inhibition is more effective in the activity control in the network than perisomatic inhibition [37]–[39]. Such a heavy involvement suggests that these interneurons are easily recruited during network activity. Early recruitment of somatostatin-expressing low-threshold spiking neurons in response to somatic depolarization in layer-5 pyramidal neurons has been reported [13]. However, the mechanisms underlying efficient activation of these dendrite-targeting inhibitory interneurons in the hippocampal CA1 region have remained unclear. The present results identify two presynaptic mechanisms that can dramatically facilitate the activation of O-LM interneurons: Activity-dependent AP broadening and static AP broadening. However, whether there are additional synaptic mechanisms underlying the marked facilitation of short-term dynamics remains to be determined. In combination, both forms of modulation markedly enhance synaptic efficacy at principal neuron–interneuron synapses, increasing EPSC amplitude substantially. Thus, both mechanisms together can promote the activation O-LM interneurons in recurrent inhibitory microcircuits.

AP modulation in the neocortex is efficiently triggered by “up” states. However, up states are lacking in the hippocampal network. Are there other rhythmic network events that may preferentially induce AP modulation? One possibility is that AP modulation occurs during theta oscillations [24], [40]. Under these conditions, slow depolarizations are generated in CA1 pyramidal neurons. Theta oscillations may induce AP modulation in two distinct ways. First, the long-lasting depolarizations during a theta cycle will enhance the frequency of burst generation, which promotes AP modulation via activity-dependent AP broadening. Second, the depolarizing theta waves will be propagated towards the presynaptic terminals directly, which generates AP modulation by depolarization-induced broadening. Thus, theta rhythm might be a rhythmic signal that effectively triggers the combination of both modulatory mechanisms. This may convert subthreshold to suprathreshold EPSPs, leading to the efficient cyclic recruitment of recurrent inhibition.

## Supporting Information

Figure S1 **Spatial spread of analog modulation in CA1 pyramidal neuron axons.** Plot of the change of axonal AP half-width by somatic depolarization as a function of the distance from the soma. Red line, exponential fit with a distance constant λ of 883 µm. (right) Examples of axonal APs measured at –60 mV (control) and –50 mV (depolarized) at 82 µm (top) and 750 µm (bottom) from the soma.
(TIF)
