# Supplementary figures and images for: Action Potential Modulation in CA1 Pyramidal Neuron Axons Facilitates OLM Interneuron Activation in Recurrent Inhibitory Microcircuits of Rat Hippocampus
Source: PLoS One. 2014 Nov 19;9(11):e113124. doi: 10.1371/journal.pone.0113124 (PMC4237399; doi:10.1371/journal.pone.0113124)

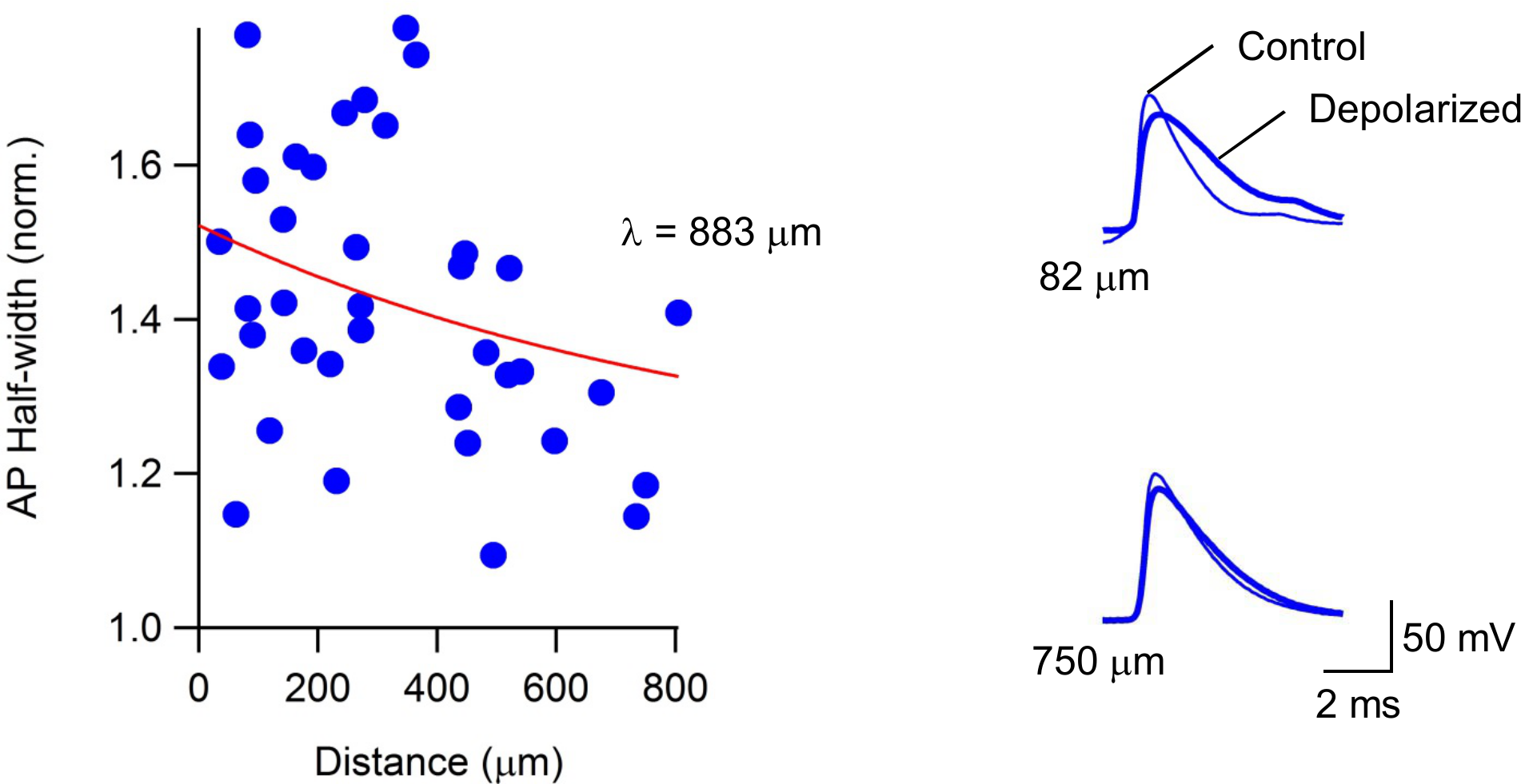

Supplement: Figure S1 — Spatial spread of analog modulation in CA1 pyramidal neuron axons. Plot of the change of axonal AP half-width by somatic depolarization as a function of the distance from the soma. Red line, exponential fit with a distance constant λ of 883 µm. (right) Examples of axonal APs measured at –60 mV (control) and –50 mV (depolarized) at 82 µm (top) and 750 µm (bottom) from the soma. (TIF) [file pone.0113124.s001.tif]
